# Why Evolve Reliance on the Microbiome for Timing of Ontogeny?

**DOI:** 10.1128/mBio.01496-19

**Published:** 2019-10-08

**Authors:** C. Jessica E. Metcalf, Lucas P. Henry, María Rebolleda-Gómez, Britt Koskella

**Affiliations:** a Department of Ecology and Evolutionary, Princeton University, Princeton, New Jersey, USA; b Department of Integrative Biology, University of California Berkeley, Berkeley, California, USA; c Department of Biology, University of Pittsburgh, Pittsburgh, Pennsylvania, USA; Yale University; University of Toronto

**Keywords:** life history evolution, microbiome, bet-hedging, host, life history evolution, ontogeny

## Abstract

The timing of life history events has important fitness consequences. Since the 1950s, researchers have combined first principles and data to predict the optimal timing of life history transitions. Recently, a striking mystery has emerged. Such transitions can be shaped by a completely different branch of the tree of life: species in the microbiome. Probing these interactions using testable predictions from evolutionary theory could illuminate whether and how host-microbiome integrated life histories can evolve and be maintained. Beyond advancing fundamental science, this research program could yield important applications. In an age of microbiome engineering, understanding the contexts that lead to microbiota signaling shaping ontogeny could offer novel mechanisms for manipulations to increase yield in agriculture by manipulating plant responses to stressful environments, or to reduce pathogen transmission by affecting vector efficiency. We combine theory and evidence to illuminate the essential questions underlying the existence of microbiome-dependent ontogenetic timing (MiDOT) to fuel research on this emerging topic.

## Abstract (precis)

The timing of life history events has important fitness consequences. Since the 1950s, researchers have combined first principles and data to predict the optimal timing of life history transitions. Recently, a striking mystery has emerged. Such transitions can be shaped by a completely different branch of the tree of life: species in the microbiome.

## OPINION/HYPOTHESIS

Many surprises have emerged as a result of our expanding knowledge of the microbiome, the community of microbial organisms that live in and on eukaryotes. Among the oddest phenomena observed is a defining role of the microbiome in the ontogeny of many host species. Key life cycle transitions, including metamorphosis in mosquitoes (1), maturation in flies (2), and flowering in plants (3), have been tied to the presence or activity of particular microbiota. This observation has been made across a wide array of ontogenetic transitions and taxa (see Table 1). Manifestations range from absolute effects (life history transitions that fail to occur in the absence of microbe species) to continuous modulations (developmental accelerations or decelerations). For example, mosquito larvae will not pupate in the absence of bacteria (1, 4), and adding bacteria or yeast rescues development (5). *Drosophila* colonized with *Acetobacter* develop more rapidly than when colonized with *Lactobacillus* (6, 7); in *Brassica*, soil microbes associated with drought led to accelerated flowering time compared to microbes associated with wet soils (8). The question then emerges: why would such an important aspect of the fitness of eukaryotic hosts be driven by the properties of organisms with very different ecologies and evolutionary histories?

For such an association to evolve, selection might have acted on the host, on the microbes, or on both. Microbes have clear incentives to manipulate host ontogeny, for example delaying transitions that result in a life stage that curtails their persistence or transmission. Host castration by parasites provides a classic and extreme example: host resources are then diverted away from host reproduction and towards parasite growth (9). In the other direction, induction of earlier flowering of Silene viscaria by the anther smut pathogen (Microbotryum violaceum) increases the probability of transmission to a new host (10).

A rich array of microbes are known to have adapted to manipulate their host’s ontogeny in these ways. Yet, the other possibility, that hosts adapt to cue into microbial signaling to determine timing of key transitions, is rarely considered. Indeed, it has been suggested that host responses to microbiome species generally reflect the outcome of chance alone (11). Yet, there is clear theoretical scope for selection to act on hosts, resulting in host dependencies on microbial signaling for key fitness-related traits. Empirical support for such effects is growing. For example, shifts in timing of flowering in *Brassica* associated with microbe presence were found to be associated with host fitness benefits (8), and squid gene expression across both circadian cycles and development has recently been linked to the presence of luminescent symbionts (12). We refer to this phenomenon as microbiome-dependent ontogenetic timing (MiDOT).

Is it worth distinguishing MiDOT from responses to classic environmental cues (e.g., rain, day length)? A focal host might be largely irrelevant to the evolutionary ecology of the microbe(s) inhabiting it (13), in which case MiDOT might resemble responses to any environmental driver. However, the ecology (and evolution) of microbes could also respond to host-related or other conditions. Host-microbe feedbacks and other dynamic changes, involving either a single microbial species or multiple species (a microbiome, where microbial interactions result in community level properties), could then importantly shift selective outcomes at the scale of the host. These processes must then be considered to understand determinants of host ontogenetic transitions.

What circumstances might lead host species to rely on cues from their microbiome (abundance/function of a single species [or group] or an outcome of the interaction of the full community [Table 1]) to trigger important life history transitions (MiDOT), rather than cues directly reflecting their own internal state or cues gleaned from the abiotic environment? Can we leverage existing tools to go beyond the correlative (host ontogeny speeds up/slows down in the presence of particular microbes) to the predictive (hosts should respond by speeding up/slowing down ontogeny in the presence of particular microbes to maximize fitness) in evaluating the role of MiDOT? Here, we describe relevant results from life history theory, outline how infectious disease ecology informs expectations for patterns of microbiome acquisition, and synthesize these threads to discuss what contexts might allow MiDOT.

**TABLE 1:** Microbiome-dependent ontogenetic timing (MiDOT) examples from host-microbe associations across terrestrial and aquatic organisms^*a*^

Host (reference)	Transition	Microbe	Transmission or mode of acquisition	Absolute or modulating effect^*b*^	Effect^*c*^
Mosquitoes (1, 4, 5)	Past 1st instar	Nonspecific	Aquatic environment	Absolute	NEI. Without microbiome, larvae do not develop past 1st instar and die.
Hydrothermal vent tubeworms (55)	Adult	Different gamma- proteobacterial species	Aquatic environment	Absolute	SMS. Larvae hatch without symbionts but acquire symbionts during settlement, losing digestive tract, surviving solely through mutualism with symbiotic bacteria.
C. elegans (52, 56)	Adult/ reproduction	*Enterobacteriaceae* species, *Comamonas*	Diet	Modulate	SMS. Different bacterial isolates can accelerate development compared to Escherichia coli.
*Drosophila* *melanogaster* (2, 47, 57, 58)	Pupation	*Acetobacter* and *Lactobacillus* species	Diet	Modulate	MOS. Sterile flies were slower to pupate than flies harboring bacteria. *Acetobacter* often accelerates, but *Lactobacillus* slows development.
Dung beetles *Onthophagus* *gazella* (59)	Pupation	Community	Brood ball	Modulate	NEI. Removal of maternally provisioned bacteria in the brood ball slows time to pupation and adult size. Soil microbes not associated with beetles do not rescue development.
*Daphnia magna* (60, 61)	Reproduction	Community	Aquatic environment	Modulate	MOS. Without microbiome, time to first egg bearing is longer compared to conventionally reared *Daphnia*. An increase in *Acidovorax* (or microbiomes enriched for) bacteria above conventionally reared *Daphnia* increases the percentage of adults bearing eggs over time.
*Arabidopsis* *thaliana* + *Brassica* * rapa* (48)	Flowering time	Community	Soil	Modulate	NEI. Experimentally evolved soil microbes for slow and fast flowering time determined flowering time in unevolved host plants.
*Brassica* * rapa* (8)	Flowering time	Community	Soil	Modulate	NEI. Drought-adapted accelerated flowering time compared to wet-adapted microbiomes in unevolved host plants, independent of drought conditions.
*Boechera* * stricta* (3)	Flowering time	Community	Soil	Modulate	NEI. Different bacterial communities determined flowering time in controlled genetic background of host plants.
Cuban tree frog *Osteopilus* * septentrionalis* (62)	Metamorphosis time	Community	Aquatic environment and other unknown sources	Modulate	NEI. Tadpoles raised in autoclaved water and long-term antibiotic treatment took twice as long to metamorphose and had lowered survival.
Turquoise killifish *Nothobranchius* * furzeri* (63)	Aging onset	Community	Aquatic environment	Modulate	NEI. Community transplant of microbiome of young fish into older fish increased life span and onset of aging.

a In these examples, experiments controlling host and microbiome variation indicate that the microbiome is a key driver in ontogenetic timing for these associations between host and environmentally acquired microbes.

b Effects can be absolute (where transition fails to occur in the absence) or modulating (where microbes speed or slow transition).

c We also indicate whether the effect can be attributed to a single microbe species (SMS), more than one species (MOS) (both of which imply construction and testing of synthetic microbiomes) or whether this was not explicitly investigated (NEI).

## PREDICTIONS FROM LIFE HISTORY THEORY

Life history theory defines how selection shapes the timing of developmental transitions (maturation, eclosion, flowering…) to maximize fitness (14). An initial important result is that delaying life history transitions is never optimal unless some benefit accrues during the delay, both because of the risk of death over the course of the delay, but also because any delay slows population growth. Beyond this, theory indicates that the optimal delay until a life history transition occurs may hinge either on endogenous features of the host (e.g., growth rate, where reproductive output is size dependent [15]) or exogenous features (e.g., temperature, time of year, where these are associated with better survival of the next life stage), or both.

**FIG 1:**
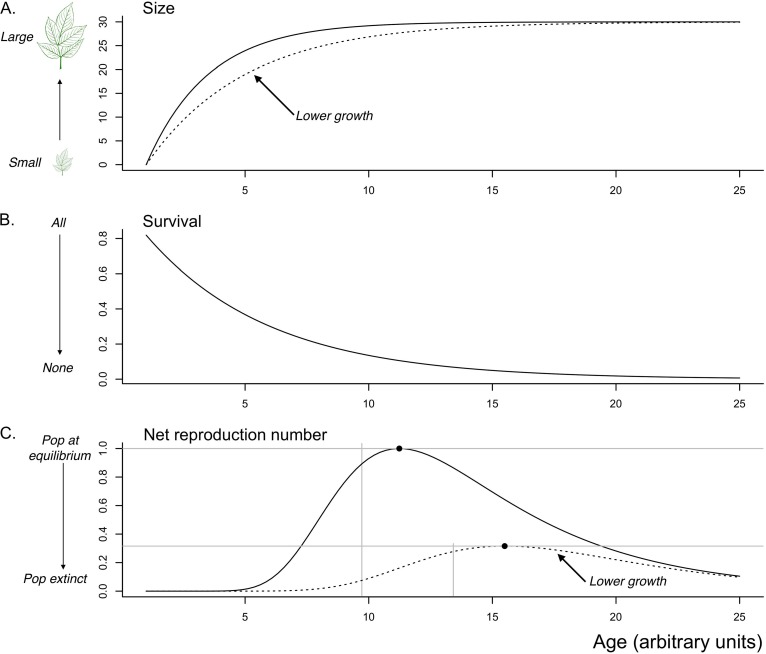
Optimal timing of monocarpic reproduction derived from different life history models. (A) Life history model where host individual size (*y* axis) saturates with age (*x* axis) following a growth function defined by *L*(*t*) = *L*_*T*_(1 − exp(−*k*(*t* − *t*_0_))) where *L*_*T*_ is the maximum possible size, *k* defines the growth rate, and *t*_0_ is the hypothetical age at which size would be zero (here, *L*_*T*_ = 30, *t*_0_ = 1, and *k* = 0.4 [solid line], or *k* = 0.25 [dashed line]). (B) Mortality occurs at rate *d*_0_, so that the probability of surviving until time *t* is *exp*(− *d*_0_*t*) (here *d*_0_ = 0.2). (C) Combining these relationships with an expression for reproductive allometries (size is converted into offspring according to *S*_*i*_ = *exp*(*A* + *BL*(*t*)); here, *A* = −5 and *B* = 1); and a probability of offspring establishment (here, *p*_*e*_ = 2 *e*^−10^ chosen to set one of the two populations at equilibrium), we can obtain an expression for the net reproduction number, *R*_0._ Since reproduction is fatal, *R*_0._ is defined by the number of offspring produced by an individual at its age of reproduction, *t*, *R*_0_ = *p*_*e*_ exp(−*d*_0_*t*)exp [*A* + *BL*_*T*_(1 − exp(−*k*(*t* − *t*_0_))))]. To identify the age at reproduction that maximizes fitness as measured by *R*_0_, we solve for *dR*_0_/*dt* = 0, which yields *t*_opt_ = *t*_0_ + log[*kBL*_*T*_/*d*_0_]/*k* (vertical lines). Pop, population.

The timing of reproduction of monocarpic species (where reproduction is fatal) provides a useful template for considering selection pressures on ontogenetic transitions. In the simplest case, theory indicates that monocarpic species (plants or animals) should reproduce when the risk of mortality over the next year outweighs the benefits accrued by delaying and growing (since larger size is associated with greater offspring production [15]) (Fig. 1). Such dependence on growth rate indicates that it is important to distinguish between scenarios where microbes are only associated with ontogenetic timing versus scenarios where microbes are associated with timing but also with other components of demography that define fitness (specifically, other relevant ontogenetic transitions or the rate of growth [2, 16] or survival [see Table 2]).

While this example (Fig. 1) illustrates the intuition underlying life history theory and shows how it can identify critical processes (e.g., microbiome species’ effects on other demographic functions), it is also unrealistic, neglecting for example the fact that for many species the environment will vary from year to year, changing the calculus associated with the costs and benefits of reproducing. However, the power of research into monocarpic species’ life history is that a set of increasingly realistic models have been developed that provide specific quantitative predictions (17, 18). Such specificity opens the way to strong tests of theory associated with MiDOT.

While much of this research has focused on optimal timing (i.e., at what age or size a transition should occur), there is also a rich set of results relating to selection for variance in timing. If individuals are selected to hedge their bets across environmental conditions that vary over time (19, 20) or if varying timing can reduce crowding and therefore competition, thus increasing fitness (21, 22), the optimal strategy requires individuals with the same genotype in the same environment to produce different phenotypes (intragenotypic variance). Seed dormancy is perhaps the best understood example of a bet-hedging trait (17); the mechanism underlying intragenotypic variation is thought to involve microgradients across seeds (23), but this does not exclude a potential role for MiDOT.

Overall, both theoretical (14, 24) and empirical (20, 25, 26) research indicates that the timing of life history transitions has important fitness consequences. Therefore, factors that accurately reflect appropriate life history timing have the potential to be harnessed as cues for ontogenetic transitions. The outcome of such selection could be MiDOT. The next questions arising to understand the selective context underlying MiDOT are therefore clear: what determines the timing of acquisition of species in the host-associated microbiome during ontogeny? Are there expectations for patterns of timing of abundance or functionality that could similarly be leveraged?

## DETERMINANTS OF THE TIMING OF MICROBIOME ACQUISITION, ABUNDANCE, AND FUNCTIONALITY

Microbiota acquisition may be vertical (transferred from parent to offspring), horizontal (microbial colonization after germination/birth/hatching from the environment or conspecifics), or a combination of both (27), and it may be mediated by host genetic variation (28, 29), amplifying the variation that selection can act upon. Acquisition of vertically transmitted microbiome species will be highly predictable (all offspring acquire their microbiome early in life). The presence of consistently vertically transmitted bacteria will thus manifest little variation that could be harnessed to structure subsequent host developmental transitions. However, changes in population growth or bacterial phenotype after initial colonization (in response to environmental cues or host size) could generate the varying time signature (or context signature) required for MiDOT to be adaptive. Thus, if vertical transmission was operating, we would expect the cue from microbial species involved in MiDOT to reflect some feature of microbial ecology, rather than microbial species’ presence alone. For example, upregulation of a microbial signaling molecule might act as a predictable signal to trigger a host developmental transition.

Considerably more variability is expected in timing of colonization for horizontally transmitted microbiome species, echoing the well-understood process of transmission for pathogens (30). In infectious disease biology, the “force of infection,” or rate at which uninfected individuals become infected (31), is defined by the product of the prevalence (of infectious individuals in the population, or of the pathogen in the environment), and the transmissibility of the pathogen (constant in the simplest case, but it could also be modulated by host age, or climatic conditions, etc.).

**FIG 2:**
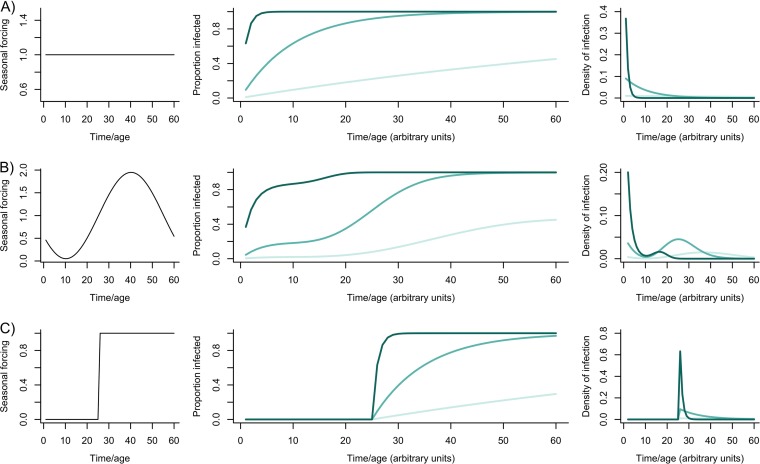
Timing information from the microbiome. For three magnitudes of the “force of infection,” or rate at which susceptible individuals are colonized by species from the microbiome (λ = 0.01, λ = 0.1, and λ = 1 colored from light to dark green, respectively), three different profiles of individuals being infected as a function of time (e.g., time during the year, or age) are obtained (middle), resulting in different patterns of age (or time) at infection (right, with increased variance for lower forces of infection). The basic patterns shown in panel A can be modulated by seasonal (B) or abrupt (C) changes in the force of infection, which can result in more or less narrowly defined age (age/timings) of infection (right).

Applying the logic of the “force of infection” to thinking about horizontal acquisition of microbiota delineates clear expectations for patterns of age- (or time-) incidence associated with particular horizontally transmitted microbiome species. If the “force of infection,” or rate of acquisition of a particular microbial species is a constant, λ, the probability of being colonized by that microbial species is 1 − exp(−λ*t*), and the average age of acquisition (or potentially timing in the year) is 1/λ (Fig. 2A); this rate also encodes variance in microbiome species’ presence across individuals in the population, defined by 1/λ^2^.

Seasonal fluctuations in the force of infection represent another key way in which a microbiome species’ presence could be used as a host cue (Fig. 2B and C). If aspects of the host affect microbiome acquisition (e.g., host behavior, body size [32]), microbiome presence could act as a predictable indicator of host age or season/timing (noting that a high rate of transmission at the appropriate stage would be required to make MiDOT reliable). Beyond the temporal or context signatures indicated above, for microbes that are dependent on hosts, nonlinear feedbacks inherent in transmission dynamics could add interesting scaling of information about population dynamics—more-rapid acquisition might indicate higher density of conspecifics, and this potentially reflects information that is relatively inaccessible to the host otherwise (see below).

## WHEN MIGHT MICROBES PROVIDE THE BEST SIGNAL AVAILABLE TO TIME TRANSITIONS?

We first focus on the situation where there is a clear optimal timing, and we evaluate three scenarios. The first scenario is that presence (presence/abundance or function) of particular microbial species reflects the most accurate signal of either the hosts’ endogenous or exogenous state in terms relevant for optimally timing an ontogenetic transition. Many environmental conditions are likely to be closely mapped by microbial species’ presence or abundance (33), given their tight ecological dependence on environmental context (e.g., pH [34], temperature [35], drought [36]). For the example of drought, there also exists the intriguing possibility that microbial species can provide nuanced information as to past environmental conditions (37), which may also be pertinent information for timing of ontogeny—legacy effects within microbial community dynamics might mean that their abundance integrates over the longer term. For example, microbes might provide a better signal of drought than seemingly more direct measures of drought like soil moisture if an overall dry season is interrupted by sporadic intense rain. Although microbial abundances vary unpredictably through time, microbial function might still be sufficiently linked to environmental conditions (38, 39) to provide useful information. Finally, one feature of hosts’ ecology which may be key to the optimal timing of ontogenetic transitions is the presence and abundance of competitors. Uniquely, species of the microbiome have the potential to provide information about this (40–42), because the “force of infection” that defines horizontal transmission (and thus the rate of acquisition of species of the microbiome) may be defined by the local abundance of hosts colonized by these microbiome species.

The second scenario is one where there are other, more-accurate signals of the relevant endogenous or exogenous state; but these are not detectable by hosts. For example, it has been suggested that plants cannot readily identify their own size, despite strong selection for size-based timing of flowering (43). If microbe acquisition (/abundance or function) maps onto the relevant timing, hosts might be selected to respond to accessible microbial cues. In general, selection to respond to a cue will depend on the quality of the cue (how reliably it predicts changes in relevant endogenous or exogenous states), but also the difficulty or cost of acquiring the cue. In many cases, even though microbes might not be the best possible source of information, they might be the most detectable: animal and plant hosts have evolved multiple receptors and regulation pathways to respond to microbial activity (44), and these can be easily coopted for ontogenetic regulation. Thus, microbes might provide reasonable information that is easy to detect.

The third scenario reflects situations where microbes modulate the conditions that shape optimal timing of flowering (e.g., by altering the growth rate [Fig. 1]). As a result, microbe species’ presence and/or abundance provides direct information as to how timing of an ontogenetic transition might be modulated to maximize fitness (3). For example in a monocarpic plant, the presence of a species that accelerates growth indicates that early flowering will be associated with higher fitness (Fig. 1C), all else being equal.

To illustrate how life history theory and microbiome ecology can be combined to develop a predictive framework, we lay out expectations for selection on MiDOT (Table 2), for the example of timing of reproduction in a monocarpic species (Fig. 1). Here, optimal timing is driven by endogenous factors, i.e., plant size (rather than exogenous factors like seasonal context), and we assume for simplicity that acquisition and abundance of the microbiome tend to increase monotonically with host age or size (but see reference 45). Organizing the ecological drivers in the context of known evolutionary selection pressures in this way (Table 2) yields clear predictions. (i) Unless the microbiome species affect a host trait other than MiDOT, only “triggering” or “increases” in the rate of the ontogenetic transition are expected. (ii) MiDOT is most likely to be associated with bet-hedging in scenarios where microbiome species only affect MiDOT. (iii) If microbiome species affect host growth, the direction of the effect will allow prediction of the expected effect of MiDOT. With more detail on particular microbiome species, or knowledge of key ontogenetic cues, such predictions could be made more specific, opening the way to formal tests of the selective value of MiDOT.

**TABLE 2:** Testable hypotheses that arise from placing microbiome-dependent ontogenetic timing (MiDOT) in a life history context^*a*^

Microbiome species effect on host life history	Possible signal		
	Presence	Abundance	Functions in specific contexts
Affects only MiDOT	Vertical transmission: no useful information (always present)	Vertical transmission: contains information if population growth is predictable; could then **trigger** or **increase** the rate of transition.	Vertical transmission: contains information if changes in microbial function are correlated with changes in host size; could then **trigger**, **increase** (or **decrease** for functions largest at small sizes) the rate of transition.
	Horizontal transmission: if no external drivers to acquisition (Fig. 2A), then high variance in timing at low transmission could potentially be leveraged for **bet-hedging**. If acquisition is context/timing specific (Fig. 2B and C), acquisition potentially selected as a **trigger** or as an **increase** in the rate of a transition.	Horizontal transmission contains information only if at high rates (low rates result in high variance [Fig. 2A, light green], where high rates have low variance, resembling vertical transmission in pattern over age/time); then selected to **trigger** or **increase** the rate of transition.	Horizontal transmission: contains information if functional succession is reliable (**trigger** or **increase** or **decrease** the rate of transition as discussed above)

Affects MiDOT **and** alters other fitness components (growth, survival); is thus guaranteed to contain a signal, since presence encodes information relevant to the optimal.	Vertical transmission: no useful information (always present)	Vertical or horizontal transmission: modulation possible (**increases** or **decreases**), see the cell above; bet-hedging unlikely; see the cell on the left	Assuming that functional composition is more important than taxonomic composition (i.e., different microbes can have the same effect on fitness components like growth and survival) then selection for **increases** or **decreases** based around functional composition expected (as discussed above).
	Horizontal transmission: effect on other fitness components might increase correlations within a cohort of hosts, thus reducing utility for bet-hedging.		
	**Increases** or **decreases** in the rate of a transition could both occur (depending on the direction of the effect on other fitness components); triggering unlikely.		

As above but in an **environment-specific** **fashion.**	As in the cell above, with the potential addition of cue indicative of specific environment.	As in the cell above, with the potential addition of cue indicative of specific environment.	As in the cell above, with the potential addition of cue indicative of environment.
Potentially makes the signal misleading if microbiome cues do not contain environmental information.			If the cue is misleading, the by-product leads to mismatch between timing and environment

a Categorizing MiDOT via its effects across the life history (leftmost column), and the information encoded by presence/abundance/functions and by-products (Possible signal columns), for vertical or horizontal transmission. We focus on the example of a monocarpic species and evaluate potential contributions to **optimizing timing** (either as a **trigger** or as **increase/decrease** in the rate of a transition [Fig. 1]) or **bet-hedging** (see the text).

## FUTURE DIRECTIONS

Characterizing if and when MiDOT is adaptive (Table 2) has potential applications from increasing agricultural yield to pathogen control. This line of research might also provide a broader physiological perspective on when the presence of particular microbiome species is more tractable and accessible to the host than direct measurement of endogenous and exogenous determinants of optimal timing, such as host size (32), or environmental conditions (20).

There is much to be done to refine our understanding of which components of a microbiome matter—from a single species to a combination of species and from their abundance to their function. In *Drosophila*, different members of synthetic microbial communities have been shown to contribute additively to timing (46, 47); in other systems, changes in community structure have been associated with MiDOT (48); but these examples are the exception, and empirical evidence is frequently lacking. Defining functional importance, whether at the strain level to bacterial species to the community level, remains a key challenge in the evolutionary ecology of microbiomes (49–51).

When the benefits of using microbiome cues outweigh the costs of potential exploitation emerging from misaligned incentives is a question ripe for detailed investigation. Taking the example of monocarpic species, delaying reproduction is likely to be desirable for any species that live on or in a host (unless vertically transmitted); increased growth in size through delayed reproduction is also likely to benefit species for whom hosts are habitat. Host manipulation by microbes for delayed reproduction then seems likely, and even tractable (e.g., where microbiome species modulate insulin pathways [2]). Alternatively, for species of the microbiota which are directly consumed by the host, accelerating development to reduce host fertility and life expectancy (as observed in Caenorhabditis elegans [52]) might be optimal. What determines the trajectories of such coevolutionary pressures? Are within-microbiome interactions sufficiently uncoordinated and competitive that they are unlikely to drive host outcomes in directions that can optimize microbiome fitness (53)? Are there situations where this is not the case? Bounding the set of contexts where MiDOT occurs and could be adaptive will open the way to evaluating the relative importance of cooption of host ontogeny.

Identifying tractable systems for probing these questions is an important step. Known host microbiome alliances (Table 1) are likely to be key. Simpler systems, including synthetic microbiomes of model systems, may also provide important insight. In the closest living relatives of animals, both the transition to multicellularity and the adoption of sexual reproduction hinge on molecules produced by bacteria in the environment, highlighting how widespread such dependencies are and providing an important potential test-bed for evolutionary predictions (33). Conversely, our overview provides few examples of MiDOT in vertebrate species, potentially as a result of more-complex buffering by the immune system (germfree vertebrates often experience developmental issues associated with immunity [54]), but it might also simply be that more data are required. Overall, the increasingly detailed resolution available on microbiome species’ effects on the biology of a diversity of hosts provides the exciting opportunity of probing the broad phylogenetic context of MiDOT and using theory and data to robustly evaluate the degree to which this surprising phenomenon is adaptive.
